# Detection of Pig Movement and Aggression Using Deep Learning Approaches

**DOI:** 10.3390/ani13193074

**Published:** 2023-09-30

**Authors:** Jiacheng Wei, Xi Tang, Jinxiu Liu, Zhiyan Zhang

**Affiliations:** State Key Laboratory for Pig Genetic Improvement and Production Technology, Jiangxi Agricultural University, Nanchang 330045, China

**Keywords:** deep learning, target detection, YOLOv8, video tracking, pig attack behavior

## Abstract

**Simple Summary:**

In this study, a deep learning-based detection and identification method is proposed to detect and identify the movement duration and aggressive behavior of pigs under on-farm conditions by using computer vision technology and electronic identity cards. The performance of different target detection algorithms for individual pig and aggressive behavior detection is also evaluated. The aim of this study is to establish an automated system for detecting pig aggressive behavior and energy expenditure, which may be able to provide reliable data and technical support for the study of the social hierarchy of pigs, as well as the selection and breeding of pig health and aggression phenotypes.

**Abstract:**

Motion and aggressive behaviors in pigs provide important information for the study of social hierarchies in pigs and can be used as a selection indicator for pig health and aggression parameters. However, relying only on visual observation or surveillance video to record the number of aggressive acts is time-consuming, labor-intensive, and lasts for only a short period of time. Manual observation is too short compared to the growth cycle of pigs, and complete recording is impractical in large farms. In addition, due to the complex process of assessing the intensity of pig aggression, manual recording is highly influenced by human subjective vision. In order to efficiently record pig motion and aggressive behaviors as parameters for breeding selection and behavioral studies, the videos and pictures were collected from typical commercial farms, with each unit including 8~20 pigs in 7~25 m^2^ space; they were bred in stable social groups and a video was set up to record the whole day’s activities. We proposed a deep learning-based recognition method for detecting and recognizing the movement and aggressive behaviors of pigs by recording and annotating head-to-head tapping, head-to-body tapping, neck biting, body biting, and ear biting during fighting. The method uses an improved EMA-YOLOv8 model and a target tracking algorithm to assign a unique digital identity code to each pig, while efficiently recognizing and recording pig motion and aggressive behaviors and tracking them, thus providing statistics on the speed and duration of pig motion. On the test dataset, the average precision of the model was 96.4%, indicating that the model has high accuracy in detecting a pig’s identity and its fighting behaviors. The model detection results were highly correlated with the manual recording results (R^2^ of 0.9804 and 0.9856, respectively), indicating that the method has high accuracy and effectiveness. In summary, the method realized the detection and identification of motion duration and aggressive behavior of pigs under natural conditions, and provided reliable data and technical support for the study of the social hierarchy of pigs and the selection of pig health and aggression phenotypes.

## 1. Introduction

Feed costs typically account for about 60% of the total cost of meat hogs. In recent years, as feed prices have continued to rise, more and more hog farms have begun to use feed equipment to evaluate the return on feed for their hogs and to improve the feed utilization of hogs using optimal linear unbiased estimation or genomic selection breeding [1,2]. Each pig typically wears a tag with an RFID electronic ear tag, and feed measurement equipment recognizes these electronic ear tags to record information such as the time of entry, weight, and feed weight for each pig. Parameters such as feed intake, body weight, and entry/exit time allow traits such as feed intake time, number of feed intakes, feed intake per meal, and feed conversion efficiency to be obtained, but the behavior and movements of the pig outside of the measurement equipment cannot be monitored. Recording the pig’s movement behavior in the whole activity area can accurately assess its daily movement trend and physical condition, which helps to select and breed excellent breeds with low energy consumption more accurately. In addition, pigs are herd animals with a stable social hierarchy within them. In general, the social hierarchy of pigs is determined by the strengths and weaknesses of group members through fights between them, and unfamiliar piglets that meet after weaning will form a social order within 48~72 h after grouping [3,4,5]. Most studies have also focused on the 48~72 h period, but the occurrence of aggressive behaviors among pigs can be observed from time to time at times greater than 72 h. Little research has been conducted in this area. Monitoring pig aggression and fighting behavior can provide insight into the social order of the herd, and because aggression consumes more energy and is more likely to result in injury, the monitoring of pig fighting has also been emphasized.

In the pig industry, farms want to select pigs with fast growth and high health; however, health is affected by pathogens and the environment, and the health status of pigs can be grasped in real-time by recording their movement attributes every day, which provides extremely detailed data for high-quality breeding pig selection. At the same time, aggression between pigs can greatly affect pork production [6], including damage to the animal’s body surface, reduced body weight and poor meat quality, and even fatal injuries [7]. In addition, stress from aggression reduces the reproductive performance of surrounding sows [8], which has a huge impact on the economic efficiency of pig farming. Therefore, aggression is considered as one of the most important health, welfare, and economic problems in modern production systems [9,10]. Studies have shown that factors such as herd management, living environment, and genetic factors are determinants of pig aggression [11]. Nevertheless, an in-depth study of pig aggression and an understanding of the factors involved will help to promote the establishment of social hierarchies in herds and reduce the daily exposure to injuries and stress, creating maximum economic value.

Currently, the behavioral identification of pigs mainly relies on manual observation in pens or under surveillance video. However, it is obvious that these methods cannot be used for long-time observation, which is not only time-consuming and laborious, but also has many limitations. In recent years, many algorithms based on deep learning techniques [12] have been developed and successfully applied to object detection tasks. Many of these algorithms use convolutional neural networks (CNNs) such as YOLOv5 and Mask R-CNN. Deep learning-based computer vision techniques are able to extract image features better and accomplish more complex tasks than traditional image-processing methods. As a result, these techniques are widely used for various detection tasks in agriculture. For example, Li et al. used the improved YOLOv5 model to recognize and count corn seedlings in a complex background and achieved 90.66% precision [13]. Li et al. proposed a lightweight convolutional neural network-based cow recognition method to train and recognize side-view images of cows, and the final recognition rate reached 97.95% [14]. Shen et al. used the YOLO model to detect cow targets in a series of side-view images of cows, classify each cow by fine-tuning the convolutional neural network model, and achieve 96.65% precision in individual cow recognition [15]. Li et al. reviewed deep learning-based methods for classification, target detection, segmentation, pose estimation, and tracking of different species of animals such as cows, goats, pigs, and poultry [16]. Guo et al. have investigated three state-of-the-art automated multi-object tracking methods on two pig datasets; the experimental results of evaluation metrics demonstrate the effectiveness and robustness of the three proposed methods on multi-object tracking systems. FairMOT, with the proposed weighted-association strategy, achieves the best tracking performance for individual pigs in a real farm [17]. Zhang et al. proposed an automated multi-target detection and tracking method for individual pigs under daytime and nighttime conditions. Overall, the evaluation resulted in a precision of 94.72%, recall of 94.74%, and MOTA of 89.58%, which shows that our method can robustly detect and track multiple pigs under challenging conditions [18]. Wutke et al. proposed a framework for the automatic detection of social contacts. By applying keypoint-based body part detection and a subsequent pig tracking algorithm, they were able to determine the time, the animals involved, and the type of a social contact [19]. The above study shows that deep learning-based computer vision technology has a wide range of promising applications in agricultural science applications: for example, it can bring more possibilities in agricultural animal production. At present, the detection and recognition of movement characteristics and aggressive behaviors of pigs are still in the beginning stage of research, and there are relatively few related research reports. One of the main reasons is that the aggression of pigs is a complex interactive behavior and can last from a few seconds to a few minutes [20]. Another feature is that aggression does not just have one form of expression. It can appear as escalated fighting, unilateral biting, pushing, non-contact assessment, etc., and animals transition between these forms of aggression rapidly. Some of these are likely to be much easier to record using deep learning than others.

We found that the geometries and displacements of two pigs in an attacking state always remain attached or at a small distance from each other, although there are abrupt changes. Therefore, we can analyze the motion of the fighting pigs as a whole [21], and identify the attacking behavior of the pigs by the overall motion characteristics. In addition, the electronic ear tags in the feed determination equipment can be effectively captured by the RF equipment, and the ID of the pig coming out of the determination equipment can be directly given to the computerized image recognition system, thus realizing the identification of the pig in the case of target loss. Therefore, this study utilizes computer vision technology in combination with an electronic ID card, adopts the improved GAM-YOLOv8-based algorithm to detect pigs and aggression, and combines it with the tracking algorithm ByteTrack [22] to track and automatically record the movement duration and aggressive behavior of pigs. Finally, the detection model and recording method were evaluated and tested by using videos and images as a test set, aiming to build a system for the automated detection of pig aggressive behavior and energy consumption.

## 2. Materials and Methods

### 2.1. Data Acquisition and Pre-Processing Methods

**Figure 1:**
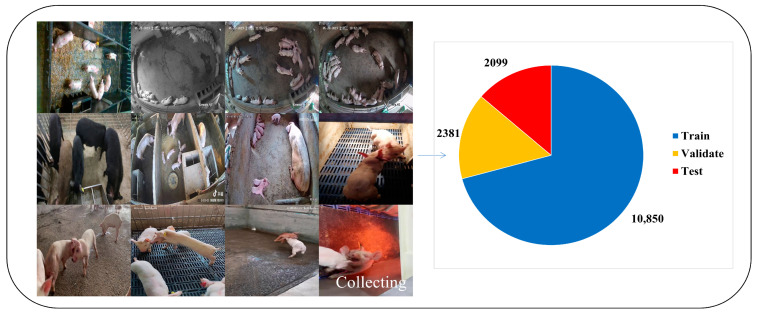
Composition of dataset.

**Figure 2:**
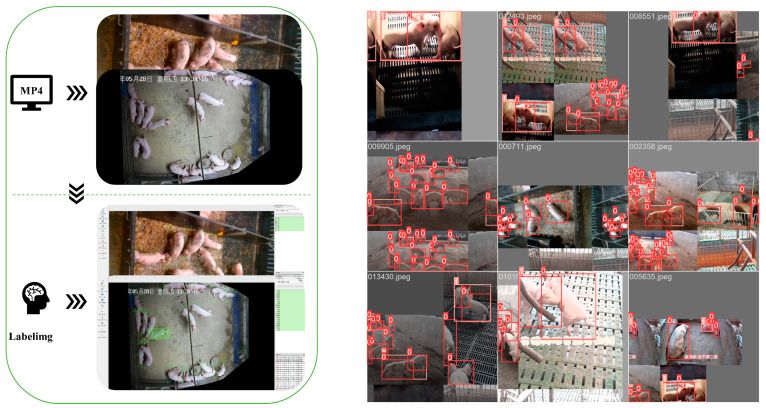
Example of data processing flow and data enhancement. Here ’0’ represents class ’pig’ in the training set and ’1’ represents class ’pig_fighting’ in the training set.

The picture and video data analyzed in this study were mainly collected from the research pig farm of the State Key Laboratory for Pig Genetic Improvement and Production Technology, Jiangxi Agricultural University. We installed a camera 3 m from the ground for filming, with a display resolution of 1920 × 1080 pixels, including a 5 m × 5 m fence, a 2 m × 5 m pen, and a 2.1 × 3.6 m farrowing bed with a leaky manure board installed on the ground. Each unit included 8~20 pigs and some of them were not familiar with each other. A smartphone, with a display resolution of 1920 × 1080 pixels, was used to collect videos and images of the pigs in the farm from different angles. To ensure the diversity of the dataset, we also collected part of the data from the web (see https://www.bilibili.com/ (accessed on 3 February 2023) and https://aistudio.baidu.com/aistudio/datasetoverview (accessed on 3 February 2023) for links). When collecting data in the network, we chose videos with lower stocking densities and higher clarity as a next step because the lack of clarity and the fact that pigs live in dense environments are not conducive to labeling. The construction and processing of the dataset are shown in Figure 1. For each video in the detection dataset, we extracted an image at every 1-frame interval. The definition of pig aggression was determined by the behavioral processes of head-to-head knocking, head-to-body knocking, neck biting, body biting, and ear biting during fighting, as determined by morphological veterinary specialists. Additionally, we need to emphasize that we define aggression when there is an obvious fight between two parties, and that an obvious fight in this case does not include situations such as a pig biting an opponent and the opponent not retaliating. On this basis, we manually labeled the aggressive behaviors in consecutive frames using LabelImg software (v1.8.6.) to produce a reference dataset of pigs and their aggressive behaviors. Ultimately, we labeled 110,701 individual pigs and 14,836 pig attacks in 15,148 images. To evaluate the model accuracy, we divided all labeled images into three datasets according to the division in Figure 1 for training, validating, and testing the detection model. An example of the data processing flow and data enhancement is shown in Figure 2. During the training process, we applied data augmentation operations such as Pan, Rotate, Flip, Blur, Add Noise, Change Contrast, Random Scaling, Random Cropping, Mosaic, etc., to prevent the model from overfitting in order to improve the generalization ability of the model, and Mosiac augmentation was turned off in the last 10 epochs, which can effectively improve the accuracy.

### 2.2. Training for Individual Identification of Pigs

**Figure 3:**
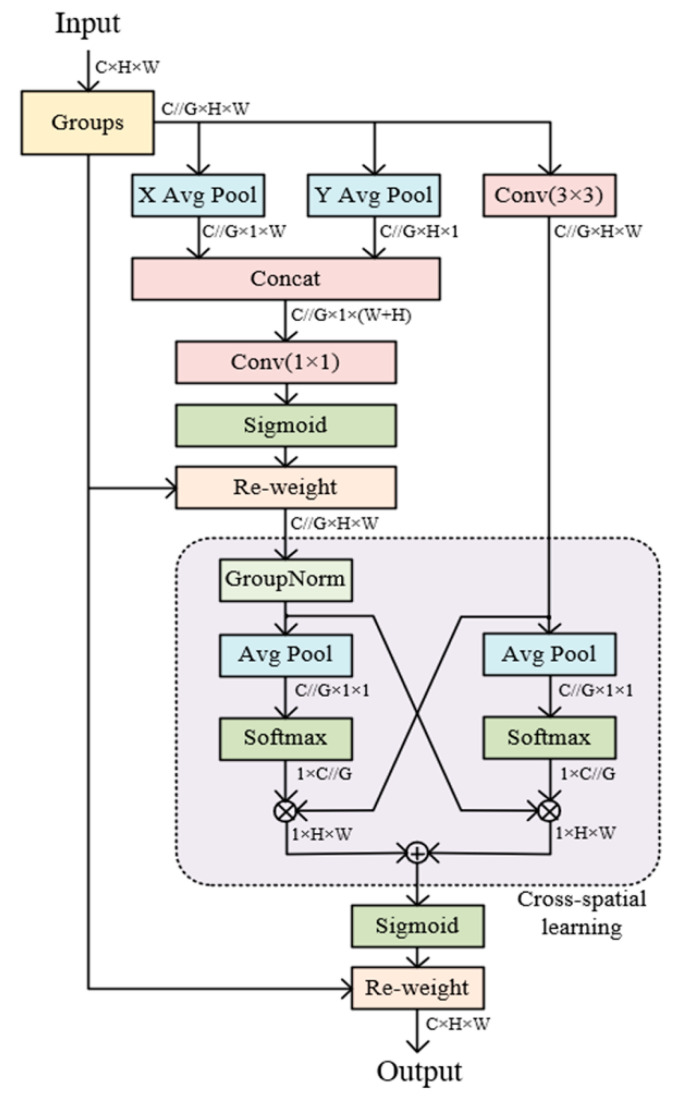
The Structure of EMA module. Avg Pool, average pooling; Conv, convolution; Sigmoid, activation function; Softmax, normalized exponential function.

**Figure 4:**
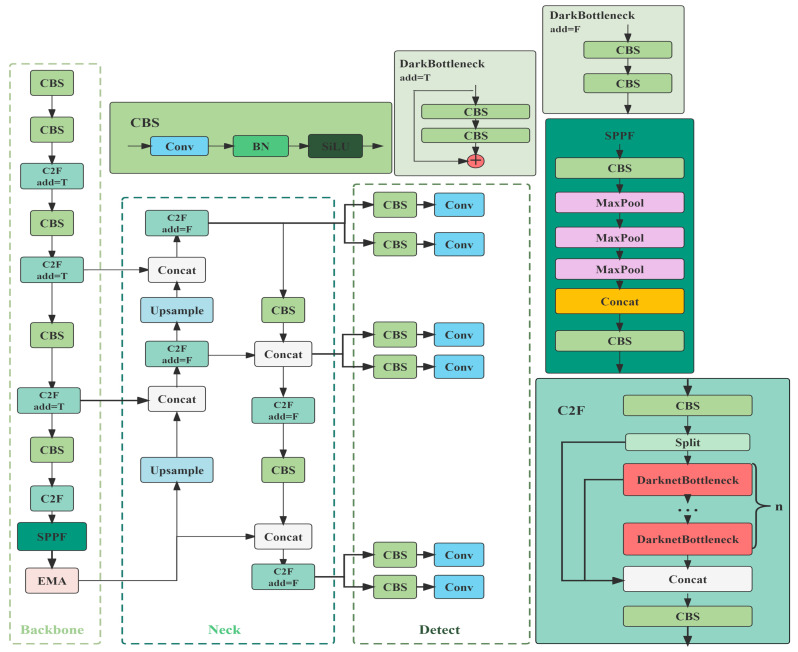
The structure of the EMA-YOLOV8n model. Conv, convolutional; BN, batch normalization; SiLU, sigmoid linear unit.

In this study, the YOLOv8 deep learning model was used for the detection of individual pigs. The initial model of YOLO had many successors, including YOLOv4, YOLOv5, YOLOv6, and YOLOv7. In 2023, YOLOv8 was released by Ultralytics [23]. YOLOv8 is the eighth generation version of the YOLO family of models, based on the latest advances in the field of deep learning and computer vision, and is recognized as the state of the art for its speed and accuracy, as it is the leading model of its kind. YOLOv8 contains five different configurations, YOLOv8n, YOLOv8s, YOLOv8m, YOLOv8l, and YOLOv8x, which gradually increase according to the depth and width of the network. The model backbone uses CSPDarknet53 [24], the head uses Decoupled Head, and the neck uses Path Aggregation Network (PANet network) [25] to maintain accuracy while realizing a lightweight design in line with advanced detection framework design concepts. In addition, it has been shown that incorporating visual attention mechanisms into deep learning models improves the recognition accuracy of small objects [26] and maintains high accuracy while being lightweight. Therefore, we introduced the multi-scale attention (EMA) module [27], the structure of which is shown in Figure 3. This module can effectively capture the information of different scales in the image, thus improving the performance of the model in multi-scale scenes. Figure 4 illustrates the structure of the improved version of EMA-YOLOV8n proposed in this study. In addition, we also use commonly used deep learning models such as Faster R-CNN, SSD, and YOLOv5 for pig-only individual recognition and attack behavior research. Faster R-CNN is an improved two-stage target detection algorithm, which has been widely used in tasks such as human pose recognition and target tracking [28]. SSD is a classical single-stage fast target detection model, which synthesizes the regression idea of YOLO and the anchor box mechanism of Faster R-CNN, and is able to achieve a good balance between detection accuracy and speed [29].

### 2.3. Tracking of Pig Motion

Dynamic objects are more difficult to track compared to static objects, especially when situations such as overlapping and occlusion occur. Compared to other tracking algorithms such as deep sort [30], ByteTrack shows significant advantages in occlusion situations.

ByteTrack is a tracking method based on the tracking-by-detection paradigm, which employs a simple and efficient data association method called BYTE. Compared with deep sort tracking algorithms, the biggest difference of ByteTrack is that instead of simply removing low-scoring detections, it utilizes the similarity between the detection frames and tracked trajectories to reduce missed detections and improve trajectory coherence. The similarity between the detection frame and the tracked trajectory, while retaining high-scoring detection results, mines the real objects (e.g., difficult samples such as occlusion and blurring) from the low-scoring detection results, thus reducing the leakage of detections and improving the consistency of the trajectory. The tracking effectiveness of the ByteTrack algorithm is heavily dependent on the accuracy of the recognition, i.e., if the detection is good, the tracking results will be improved, but if the detection is poor, the tracking results will be severely affected. Specifically, the flow of the BYTE data association method is as follows:

- Separate the detection frames into high- and low-scoring frames based on the detection frame score.
- The first time, the high score box is used to match the previous trace.
- The second time, we use the low-scoring frame to match the track that did not match the high-scoring frame the first time (e.g., objects that were heavily occluded in the current frame that caused the score to drop).
- Create a new track for frames that do not match the track but have a high enough score. Tracks that do not match a frame are kept for 30 frames and matched when they appear again.

**Figure 5:**
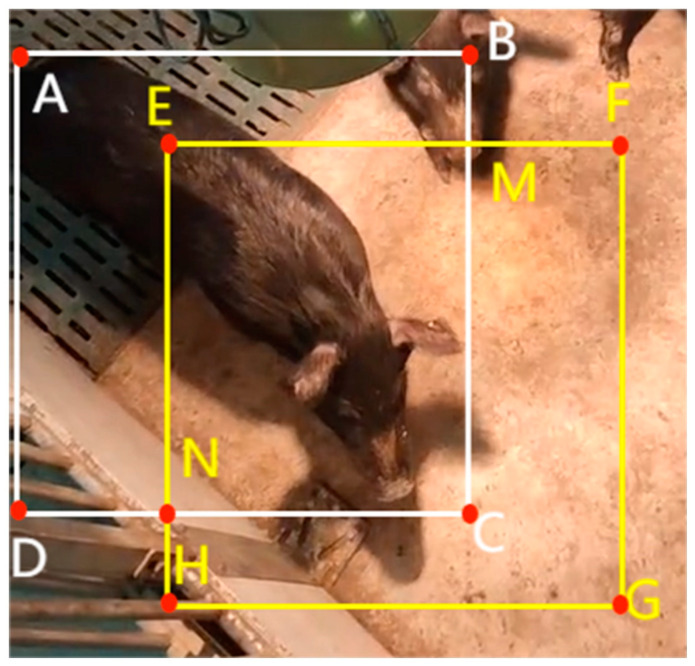
A detection box and a tracker for a pig.

In addition to this, the method uses Kalman filtering to predict the position of the tracking trajectory of the current frame in the next frame, and the IoU (Intersection over Union) between the predicted frames and the actually detected frames is used as the similarity between the two matches when the matching is carried out via the Hungarian algorithm. Figure 5 shows a schematic diagram demonstrating how the tracking system detects the pigs and uses the bounding box to track their positions. The IoU metric is used to measure the overlap between the predicted frames of the tracker and the detector, and is computed as shown in Equation (1), where S_EMCN_, S_ABCD_, and S_EFGH_ denote the areas of the rectangles in Figure 5, respectively.

(1) $$\mathrm{IoU}=\frac{s_{\mathrm{EMCN}}}{s_{\mathrm{ABCD}}+S_{\mathrm{EFGH}}-S_{\mathrm{EMCN}}}$$

### 2.4. Detection and Tracking of Aggressive Behavior and Movement Trajectories in Pigs

**Figure 6:**
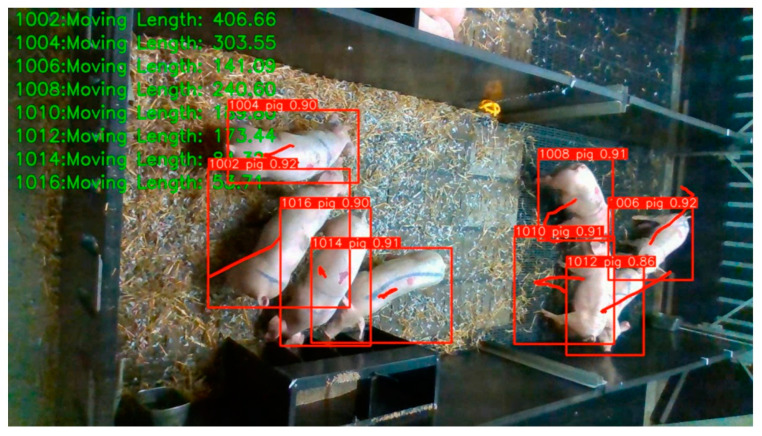
The schematic diagram of pig movement recording.

In actual production, each pig has a unique electronic ear number and can be recognized by feed determination equipment. During the tracking process, the ID of each pig obtained using video recognition is matched with the digital ID of its electronic ear tag, which facilitates the correspondence between the feeding phenotype and the pig’s ear number, and, at the same time, if the video recognition loses the target, the association can be re-established by the feed determination equipment. For each recognized pig, the corresponding digital ID, category, and coordinates of the detection frame in each frame are extracted, and the position of the center point of the detection frame is calculated. Then, lines are drawn between the center points of the detection frames with the same digital ID, and the distance between the current frame and the center point position of the previous frame is calculated based on the coordinates and accumulated to obtain the motion distance of the pig with the corresponding ID, as shown in Figure 6 and Supplementary Video S1.

**Figure 7:**
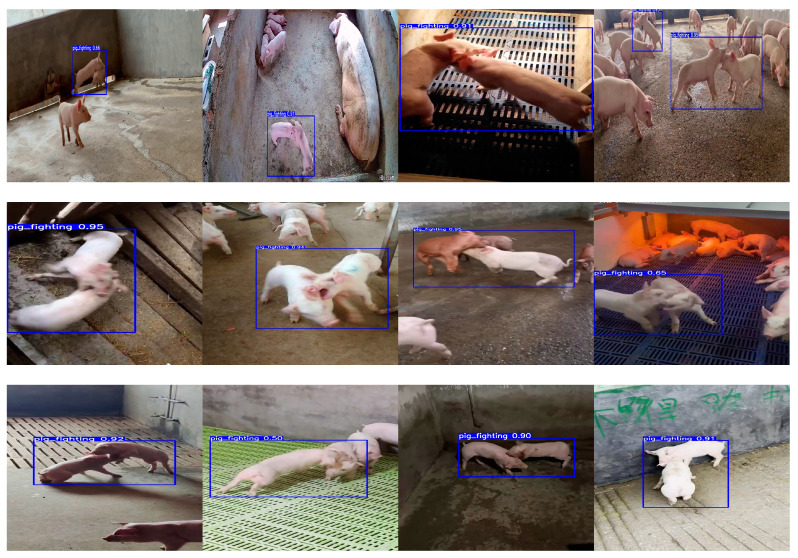
Example of a pig attack detection box.

When the pig attack behavior detection box appears, the current point in time is noted as T1; when the attack behavior detection box disappears, the current point in time is again noted as T2. Setting up the output of T1, T2, and the current ID number given to the background file realizes the recording of the time of the pig fights for the subsequent analysis of the pig’s behavior. Figure 7 shows the detection box of the attack behavior.

### 2.5. Model Training and Test Precision Evaluation

In order to evaluate the recognition and detection performance of the model, we evaluated the detection performance of the model in terms of precision, recall rate, mean average precision (*mAP*), and floating point operations (FLOPs) as per the following metrics:

(2) $$Precision=\frac{TP}{TP+FP}$$

(3) $$Recall=\frac{TP}{TP+FN}$$

(4) $$mAP=\frac{\sum_{1}^{n}\int_{0}^{1}Precison\left(Recall\right)d\left(Recall\right)}{n}$$

where *TP*, *FP*, and *FN* are the number of true positives, false positives, and false negatives, respectively. In Equation (4), AP is the area under the exact recall curve (P–R curve) and *mAP* is the average of different categories of AP. mAP_@0.5_ represents the mean value of *mAP* when the IOU threshold is 0.5. The operating system used for model training and testing in this study is Windows 10; the versions of Python and Pytorch used for deep learning are 3.9 and 2.0.0, respectively; the CPU and GPU used are Inter I7-13700K and Nvidia GeForce RTX 4070Ti, respectively; the CUDA (Compute Unified Device Architecture) and CUDA deep neural network library are 11.8 and 8.7.0, respectively; the CPU and GPU (Graphics Processing Unit) used are Inter I7-13700K and Nvidia GeForce RTX 4070Ti, respectively; and the CUDA (Compute Unified Device Architecture) and CUDA deep neural network libraries are 11.8 and 8.7.0, respectively. During the training process, the batch sizes and epochs were 32 and 100, respectively, and all other parameters were recommended by the official website.

## 3. Results

### 3.1. Setting of Parameters of the YOLOV8 Model

In order to select a suitable model for pig detection, we trained and tested three configurations of YOLOv8 (n, s, and m series), while the l and x series models were not taken into account due to the excessive number of parameters. The results of the comparison of each model on the test set are shown in Table 1. From the table, it can be observed that as the complexity of the YOLOv8 model increases, the mAP_@0.5_ for both the pig class and the pig_fighting class shows an upward trend, but the corresponding number of parameters and floating-point operations (FLOPs) also increases. Specifically, YOLOv8n has a slightly lower mAP_@0.5_ of 0.6% for the pig class relative to YOLOv8s and YOLOv8m; the mAP_@0.5_ for the pig_fighting class is only slightly lower than YOLOv8m’s 0.7%. In addition, the number of parameters of YOLOv8n is reduced by 8.12 M and 22.83 M compared to YOLOv8s and YOLOv8m, respectively; meanwhile, its FLOPs are reduced by 20.3 G and 50.5 G compared to YOLOv8s and YOLOv8m, respectively. Therefore, under the comprehensive consideration of the detection accuracy and the model parameters, we chose YOLOv8n as the detection model and further improved it on this basis.

### 3.2. Improvements in Different Attention Mechanisms in Model Training

In order to improve the detection accuracy while achieving a light weight, we improved the YOLOv8n model. Meanwhile, in order to verify the performance advantage of the EMA (Exponential Moving Average) module in this experiment, we combined EMA with other attention mechanism modules, such as Global Attention Mechanism (GAM) [31], Squeeze-and-Excitation (SE) [32], and Convolutional Block Attention Module (CBAM) [33]. They were each combined at the same location and evaluated on the same test set. The comparative results of the improved models are shown in Table 2. The model incorporating the EMA module demonstrated higher accuracy for detecting pig identity and pig aggression compared to the other attention mechanisms. Relative to the previous YOLOv8m model with the best performance in detection accuracy, EMA-YOLOv8n was only slightly lower by 0.1% in mAP_@0.5_ for pig classes, while the number of parameters and FLOPs remained consistent with the smallest YOLOv8n model. Therefore, we chose the EMA-YOLOv8n model for the detection and identification of pig identities and aggressive behaviors.

### 3.3. Evaluation of Different Models for Detection of Pig and Aggressive Behavior

In order to validate the ability to detect pig aggression, we evaluated each model using images from the test dataset. Table 3 shows the results of different training models in the evaluation. As shown in the table, the mAP_@0.5_ of EMA-YOLOv8n is much better than that of Faster R-CNN, and its number of parameters and FLOPs is much smaller than that of Faster R-CNN. The reason for the lower mAP_@0.5_ of Faster R-CNN may be due to the presence of occlusion during pig detection, while Faster R-CNN is not amenable to occluded objects. Compared to SSD, the mAP_@0.5_ of EMA-YOLOv8n in detecting pig class is 2.0% higher, and its number of parameters and FLOPs are reduced by 20.86 M and 335.7 G, respectively. In addition, the mAP_@0.5_ of YOLOv5n in detecting pig class and pig_fighting class is slightly lower than that of EMA-YOLOv8n by 0.2% and 1.7%, respectively, which indicates that EMA-YOLOv8n has a higher detection accuracy. Therefore, considering the detection accuracy and model parameters, we chose the EMA-YOLOv8n model for the detection and identification of pig identities and aggressive behaviors in practical applications.

### 3.4. Evaluation of Model Generalization Capability

**Figure 8:**
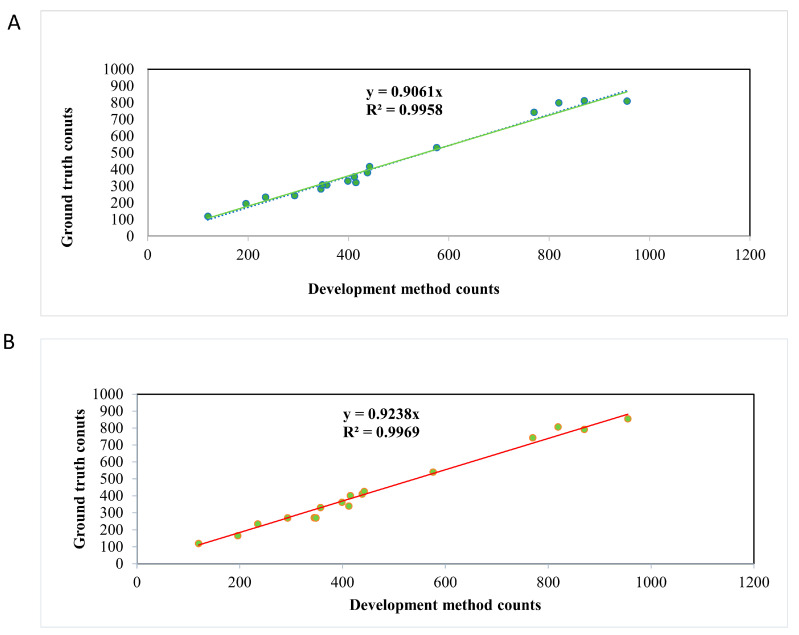
Results of artificial recognition algorithm and regression analysis. (**A**) Regression analysis to identify pigs. (**B**) Regression analysis to identify pig aggression.

To evaluate the generalization ability of the model, we selected 18 video clips with different angles and that were completely different from those of the pigs in the training set, and compared the recognition results of the model algorithm with those of manual recognition. We calculated the number of frames in which the algorithm correctly identified pigs and pig aggression versus the number of frames correctly identified manually, and performed a regression analysis. Figure 8A shows that the results of the algorithmic identification of pigs were highly similar to the manual identification of pigs (R^2^ = 0.9804). Figure 8B also shows the same results for the algorithmic recognition of pig aggression versus the manual recognition of pig aggression (R^2^ = 0.9856), which suggests that the detection and recognition results of the model algorithm have potential applications in pig and aggression detection.

## 4. Discussion

In order to efficiently detect the motion behaviors of pigs, this study adopts a deep learning target detection algorithm and tracking algorithm to construct a model for detecting the motion behaviors of pigs, and realizes the tracking of the motion trajectory and the statistics of the motion duration of pigs by assigning a fixed electronic numerical ID to the pigs. Currently, the study of social hierarchies in pigs and the improvement in their welfare has attracted a lot of attention. It has been found that when weaned piglets are mixed, intense fighting among piglets occurs due to changes in group membership to establish a new social hierarchy [3]. Stookey et al. showed that fighting behavior after mixing may reduce food conversion efficiency and affect piglet weight gain [6]. Li and Johnston et al. found that social hierarchy re-establishment affects weaned piglets’ fighting behavior and productive performance [34]. In pig farming, aggression is considered to be one of the most important health and welfare issues in modern production systems, and may lead to adverse effects including damage to the animal’s body surface, weight loss, and even fatal injuries. However, current research on social hierarchy focuses on 48 to 72 h after mixing, and there are fewer studies on the interaction behaviors among pigs after 72 h. Monitoring pig aggression for a longer period of time can provide a more in-depth study of the social order of the herd, but it is currently usually carried out using video footage or manual observation. At the same time, if pigs can be selected for less fighting by the phenotypic detection of pig aggression, then animal welfare will potentially improve.

In this study, we propose a deep learning-based method for detecting and recognizing pig motion durations and aggressive behaviors, and evaluate the performance of different target detection algorithms for individual pig identification and aggressive behavior detection using computer vision techniques and electronic identity cards, combined with video and image datasets. Subsequently, we adopted the improved EMA-YOLOv8n model and combined it with the tracking algorithm ByteTrack to achieve the real-time and efficient detection and recognition of pig movement and aggressive behavior. Notably, the results of the model generalization ability test show that our proposed detection method is highly similar to the manually recorded data in recognizing pig identity and aggressive behaviors (R^2^ values of 0.9804 and 0.9856, respectively). We also hoped that our method would be able to detect less obvious (i.e., less escalating) forms of aggression, such as single bites that do not result in obvious bilateral grappling; however, our model is much stricter in its determination, and there are ambiguous behaviors that we do not determine as fights. We are well aware that this type of less obvious aggression can also lead to long-term social stress, which can affect, among other things, productivity and well-being, and we are therefore keen to see our method help other researchers to better address this issue as well.

## 5. Conclusions

In summary, in this study, we proposed a deep learning-based detection and recognition method for pig motion duration and aggressive behavior, and evaluated the performance of different target detection algorithms for individual pig and aggressive behavior detection by using computer vision techniques and electronic identity cards, combined with video and image datasets. Meanwhile, we proposed a novel EMA-YOLOv8n model and combined it with the tracking algorithm ByteTrack to realize the real-time and efficient detection and recognition of pig motion and aggressive behaviors, as well as the tracking of pig motion trajectories, which led to the statistics of pig motion duration. In addition, the results of the model generalization ability test show that our proposed detection method is highly similar to the manually recorded data in recognizing pig and aggressive behaviors (R^2^ values of 0.9804 and 0.9856, respectively). Therefore, the proposed modeling method provides an effective way to achieve the detection and identification of pig motion and aggressive behaviors. In addition, the detection and annotation images used in this study can provide a useful reference for other researchers to further explore methods for recognizing, recording, and analyzing pig behaviors.

## Figures and Tables

**Table 1:** The comparison results of the YOLOV8 (n, s, and m series) modes.

Models	Class	mAP_@0.5_ (%) ^1^	Parameters	FLOPs (G)
YOLOV8n	Pig ^2^	95.6	3.01 M	8.1
	pig_fighting ^3^	95.9		
YOLOV8s	pig	96.2	11.13 M	28.4
	pig_fighting	95.9		
YOLOV8m	pig	96.2	25.84 M	78.7
	pig_fighting	96.6		

^1^ Mean average precision; ^2^ the object of detection in the datasets is pig; ^3^ the object of detection in the datasets is the aggressive behavior of pigs.

**Table 2:** The comparison results of adding different attention mechanisms.

Models	Class	mAP_@0.5_ (%) ^1^	Parameters	FLOPs (G)
YOLOV8n	Pig ^2^	95.6	3.01 M	8.1
	pig_fighting ^3^	95.9		
SE-YOLOV8n	pig	95.8	3.01 M	8.1
	pig_fighting	95.8		
CBAM-YOLOV8n	pig	96.0	3.01 M	8.1
	pig_fighting	95.9		
EMA-YOLOV8n	pig	96.1	3.02 M	8.1
	pig_fighting	96.6		
GAM-YOLOV8n	pig	96.1	4.65 M	12.5
	pig_fighting	96.2		

^1^ Mean average precision; ^2^ the object of detection in the datasets is pig; ^3^ the object of detection in the datasets is the aggressive behavior of pigs.

**Table 3:** The posed detection model and comparison results of pigs and aggressive behavior.

Models	Class	mAP_@0.5_ (%) ^1^	Parameters	FLOPs (G)
SSD	Pig ^2^	94.1	23.88 M	343.3
	pig_fighting ^3^	96.4		
Faster R-CNN	pig	52.4	41.13 M	193.78
	pig_fighting	46.5		
YOLOV5n	pig	95.9	1.76 M	4.1
	pig_fighting	94.9		
EMA-YOLOV8n	pig	96.1	3.02 M	8.1
	pig_fighting	96.6		

^1^ Mean average precision; ^2^ the object of detection in the datasets is pig; ^3^ the object of detection in the datasets is the aggressive behavior of pigs.

## Data Availability

The data presented in this study are available on request from the corresponding authors.

## Supplementary Materials

The following supporting information can be downloaded at https://www.mdpi.com/article/10.3390/ani13193074/s1, Supplementary Video S1. Pigs were detected, tracked, and assigned an artificially assigned fixed numeric ID, in a video sequence comprised of successive frames. A red bounding box represents a pig that was detected in the current frame. The red line represents the trajectory, and, beyond a certain distance, the front part of the track line disappears. In the upper left corner of the video is the motion distance of the pig. For demonstration, we recorded the motion distances of the pigs with ID 1002 and 1004.
